# DISC1 mutant macaques capture behavioral and neural hallmarks of psychiatric disease

**DOI:** 10.1093/nsr/nwag200

**Published:** 2026-03-28

**Authors:** Wenjia Zhou, Jiayu Sun, Guo-Long Zuo, Yijun Cai, Yang Li, Puzhe Li, Xinyue Tan, Chengfeng Wu, Runze Zhang, Ling Zhuang, Xiaochun Wang, Yanhong Nie, Yan Wang, Wen Fang, Chengyu Li, Zheng Wang, Ji Hu, Hung-Chun Chang, Zhiqi Xiong, Ian Max Andolina, Liping Wang, Qiang Sun, Zhen-Ge Luo

**Affiliations:** School of Life Science and Technology, ShanghaiTech University, Shanghai 201210, China; Institute of Neuroscience, Center for Excellence in Brain Science and Intelligence Technology, Chinese Academy of Sciences, Shanghai 200031, China; School of Life Science and Technology, ShanghaiTech University, Shanghai 201210, China; Institute of Neuroscience, Center for Excellence in Brain Science and Intelligence Technology, Chinese Academy of Sciences, Shanghai 200031, China; Institute of Neuroscience, Center for Excellence in Brain Science and Intelligence Technology, Chinese Academy of Sciences, Shanghai 200031, China; School of Life Science and Technology, ShanghaiTech University, Shanghai 201210, China; School of Life Science and Technology, ShanghaiTech University, Shanghai 201210, China; Institute of Neuroscience, Center for Excellence in Brain Science and Intelligence Technology, Chinese Academy of Sciences, Shanghai 200031, China; Lingang Laboratory, Shanghai 201103, China; School of Life Science and Technology, ShanghaiTech University, Shanghai 201210, China; Institute of Neuroscience, Center for Excellence in Brain Science and Intelligence Technology, Chinese Academy of Sciences, Shanghai 200031, China; Institute of Neuroscience, Center for Excellence in Brain Science and Intelligence Technology, Chinese Academy of Sciences, Shanghai 200031, China; University of Chinese Academy of Sciences, Beijing 100049, China; Institute of Neuroscience, Center for Excellence in Brain Science and Intelligence Technology, Chinese Academy of Sciences, Shanghai 200031, China; Institute of Neuroscience, Center for Excellence in Brain Science and Intelligence Technology, Chinese Academy of Sciences, Shanghai 200031, China; Institute of Neuroscience, Center for Excellence in Brain Science and Intelligence Technology, Chinese Academy of Sciences, Shanghai 200031, China; Institute of Neuroscience, Center for Excellence in Brain Science and Intelligence Technology, Chinese Academy of Sciences, Shanghai 200031, China; Institute of Neuroscience, Center for Excellence in Brain Science and Intelligence Technology, Chinese Academy of Sciences, Shanghai 200031, China; Lingang Laboratory, Shanghai 201103, China; School of Psychological and Cognitive Sciences, Beijing Key Laboratory of Behavior and Mental Health, State Key Laboratory of General Artificial Intelligence, IDG/McGovern Institute for Brain Research, Peking‐Tsinghua Center for Life Sciences, Peking University, Beijing 100871, China; School of Life Science and Technology, ShanghaiTech University, Shanghai 201210, China; Lingang Laboratory, Shanghai 201103, China; School of Life Science and Technology, ShanghaiTech University, Shanghai 201210, China; Institute of Neuroscience, Center for Excellence in Brain Science and Intelligence Technology, Chinese Academy of Sciences, Shanghai 200031, China; University of Chinese Academy of Sciences, Beijing 100049, China; Shanghai Center for Brain Science and Brain-Inspired Technology, Shanghai 201602, China; Institute of Neuroscience, Center for Excellence in Brain Science and Intelligence Technology, Chinese Academy of Sciences, Shanghai 200031, China; Institute of Neuroscience, Center for Excellence in Brain Science and Intelligence Technology, Chinese Academy of Sciences, Shanghai 200031, China; Institute of Neuroscience, Center for Excellence in Brain Science and Intelligence Technology, Chinese Academy of Sciences, Shanghai 200031, China; School of Life Science and Technology, ShanghaiTech University, Shanghai 201210, China; State Key Laboratory of Advanced Medical Materials and Devices, ShanghaiTech University, Shanghai 201210, China

**Keywords:** neurological disorders, *DISC1*, non-human primate, synaptic dysfunction, comorbidity, fluoxetine

## Abstract

Schizophrenia is a devastating and complex neurological disorder with poorly understood neurodevelopmental origins. Current rodent models often fail to fully capture human symptomology, hindering therapeutic development. We generated a germline-transmissible *DISC1* mutant model in cynomolgus macaques using CRISPR-Cas9 targeting of exon 9. F0 founders and their F1 heterozygous offspring displayed increased stereotypic behaviors and self-injury, reduced exploration, social withdrawal, and sleep fragmentation. Fluoxetine partially ameliorated these behaviors in one founder. Neuroimaging revealed enlarged dorsal striatum (suggesting dopaminergic hyperfunction) and reduced medial amygdala (associated with emotional dysregulation). Plasma metabolomics indicated elevated dopamine and 3-methoxytyramine alongside decreased serotonin metabolite hydroxyindoleacetic acid (HIAA). Notably, one mosaic male exhibited enhanced visual precision and aberrant multisensory integration. Single-nuclei RNA sequencing of the dorsolateral prefrontal cortex revealed an excitatory/inhibitory imbalance—specifically, reduced parvalbumin-positive interneurons and synaptic dysregulation—accompanied by downregulation of autism-risk genes. These macaques recapitulate key psychiatric phenotypes including social deficits, aggression and anxiety, providing a valuable model for screening and testing of targeted therapeutics.

## INTRODUCTION

Neurological disorders represent significant global health challenges, contributing substantially to both medical burdens and economic costs. The Global Burden of Diseases, Injuries, and Risk Factors Study (GBD) 2019 indicated that ∼12% of the global population experienced a neurological disorder in 2019, with depressive and anxiety disorders among the most prevalent conditions [1]. Increasingly, research suggests that neuropsychiatric illnesses such as major depressive disorder (MDD) and schizophrenia (SZ) arise from complex interactions between genetic vulnerability and environmental influences [2]. Critically, symptom overlap is frequently observed across these disorders, alongside high rates of comorbidity—for example, anxiety-depression co-occurrence or the presence of depressive symptoms within SZ presentations [3,4].

*DISC1* encodes a scaffolding protein that interacts with multiple synaptic and cytoskeletal molecules, playing essential roles in synaptic plasticity, neuronal proliferation, migration, and differentiation [5]. A translocation (1;11) (q42.1; q14.3) disrupting *DISC1* was identified in a Scottish family, where it co-segregated with major psychiatric disorders, including SZ, MDD, and autism spectrum disorder (ASD) [6,7]. Subsequent studies have generated numerous DISC1 mutant mouse models, which exhibit a range of anatomical, neurochemical, and behavioral abnormalities. Common phenotypes in these models include dendritic spine loss, reduced parvalbumin-positive (PV^+^) interneurons, sensorimotor gating deficits, and impaired sociability. Further investigations using *DISC1*-mutant human induced pluripotent stem cells (iPSCs) revealed synaptic dysregulation, impaired cortical neuronal subtype specification, and GABAergic dysfunction in both forebrain neurons and organoids [8–11]. While these models have provided valuable insights into the pathophysiology of major psychiatric disorders at the molecular level, they failed to fully capture the higher cognitive impairments and complex social behaviors observed in patients [12]. A major challenge in translational psychiatry lies in bridging the gap between rodent/human iPSC-based models and clinically relevant medical interventions. Given these limitations, non-human primates (NHPs) are essential intermediate model system for studying psychiatric disorders. NHPs serve as highly relevant models for neuroscience research due to their close resemblance to humans in brain organization, social behavior, sensory processing, and genetic makeup [13–15]. The progress of CRISPR-Cas9-mediated gene editing has facilitated the development of targeted gene-disrupted NHP models [16]. Previous studies have established *MECP2*-, *SHANK3*-, and *BMAL1*-edited NHPs as valuable models for ASD and psychiatric conditions [17–21]. In this study, we generated a *DISC1*-mutant cynomolgus macaque (*Macaca fascicularis*) model and characterized its behavioral and neurobiological phenotypes. The mutant monkeys exhibited significant alterations in sleep patterns, increased stereotypic behaviors, self-injurious tendencies and impaired social interactions, accompanied by structural brain abnormalities and neuroendocrine dysregulation. Single-nucleus RNA sequencing (snRNA-seq) analysis revealed disruptions in excitatory/inhibitory neuronal balance and synaptic function. Notably, pharmacological intervention with fluoxetine in one *DISC1*-mutant macaque ameliorated stereotypic and self-injurious behaviors, supporting the utility of this model for both mechanistic studies of mental disorders and preclinical drug development. These findings collectively demonstrate the translational potential of *DISC1*-mutant NHPs for future neuropsychiatric research and targeted development of therapeutics.

## RESULTS

### Germline-transmissible DISC1 mutation in macaques

To generate *DISC1*-mutant cynomolgus macaques, we employed a CRISPR-Cas9 strategy targeting exon 9 of the macaque *DISC1* gene. Cas9 mRNA and three single-guide RNAs (sgRNAs) were microinjected into macaque embryos, and editing efficiency was confirmed (Fig. S1A). A total of 93 injected embryos were transferred into surrogate females, yielding nine live births, of which four (M1–M4) survived beyond three years, while five died within six months (Fig. S1B and C, Table S1). As previously reported [16], CRISPR-Cas9 editing induced mosaicism with diverse insertion-deletion (indel) mutations (Fig. S1D). M2 exhibited complete mutant mosaicism (CM), with no detectable wild-type (WT) *DISC1* alleles. In contrast, M1, M3, and M4 were incomplete mosaics (IM), carrying ∼70% (M1, M4) or 40% (M3) mutated alleles, including deletions (–), insertions (+), and point mutations (PM). Most indels introduced frameshifts and early stop codons near the sgRNA target sites, except for one 60-bp deletion, which resulted in a 20 aa truncation. Genotyping of cortex, cerebellum, muscle, liver, and skin from aborted *DISC1*-mutant macaques confirmed widespread CRISPR-Cas9 editing across endodermal, mesodermal, and ectodermal lineages (Fig. S1E).

To assess germline transmission of the DISC1 mutation, we performed single-sperm injection (collected from M3) into WT oocytes. Genotyping of 10 embryos revealed the +5 bp indel at a frequency (∼30%) comparable to that observed in M3 somatic cells (Fig. S1C). Following embryo transfer, we obtained five live F1-generation macaques and seven aborted fetuses. In the F1 generation, we identified two macaques (HET1, HET2) carrying the +5 bp indel alongside WT alleles (Fig. S1F and G). These animals represent heterozygous *DISC1* mutants without genetic mosaicism, confirming successful germline transmission. We confirmed the decreased expression of DISC1 protein in F0 and F1 generations. (Fig. S1H). Potential off-target sites were predicted using Cas-OFFinder (http://www.rgenome.net/cas- offinder/) [22] (Table S2). We analyzed the 19 off-target sites (at introns or exons) in 17 *DISC1*-mutant macaques using sanger sequencing and the alignment analysis revealed that only M6 had a 5 bp insertion at 200 bp near the potential off-target site at intron of *ALG14* (Tables S3 and S4). M6 is an aborted *DISC1* mutant macaque that was not used in the following analysis.

We monitored general health, including heart rate and body weight, monthly in *DISC1*-mutant macaques and age- and sex-matched WT controls from the same colony during their first two years. While body size and weight of mutant macaques fell within the normal range for the species during this period, M1 and M2 exhibited smaller body sizes than WT macaques from their third year onward. By 45 months of age, M1 and M2 showed reduced body weight, whereas M3 remained comparable to WT controls (Fig. S1I and J). The two surviving F1 heterozygous macaques (HET1 and HET2) displayed normal body development in their first postnatal year (Fig. S1J). Thus, combined delivery of Cas9 mRNA and three guide RNAs (sgRNAs) targeting exon 9 effectively mediates *DISC1* editing in macaques, and this mutant allele demonstrates germline transmissibility to subsequent generations.

### Abnormal stereotypic and self-injurious behavior in home cage and novel environment

Patients with major neurological illness frequently experience recurrent symptom episodes characterized by reduced motivation, social withdrawal [23,24], deficits in executive function and memory [25], and heightened anxiety accompanied by exaggerated responses to unexpected stimuli [26]. Notably, lower *DISC1* mRNA levels have been associated with the emergence of positive, negative, and depressive symptoms in human patients [27]. To investigate DISC1-associated phenotypes in our NHP model, we conducted a series of behavioral tests at multiple time points for F0 founder *DISC1* mutant monkeys and at 18 months (18 M) for the F1 generation [28].

**Figure 1.**
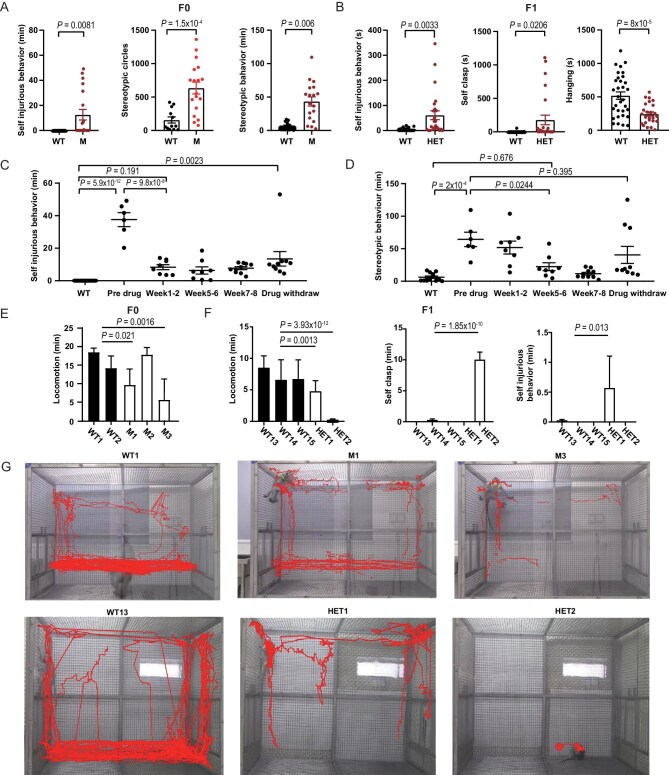
DISC1-mutant macaques display abnormal behaviors. (A) Stereotypic and self-injurious behaviors of F0 monkeys in their home cage during the daytime (12 h). (WT, *n* = 12, M, *n* = 18). (B) Self-injurious, self-clasp and hanging behaviors in the home cage in F1 monkeys (WT, *n* = 33, HET, *n* = 25). Two-tailed Welch’s *t*-test. (C and D) Continuous recording of M3 in home cage for self-injurious and stereotypic behaviors before and after taking fluoxetine (WT, *n* = 12; pre drug, *n* = 6; Week 1–2, *n* = 8; Week 5–6, *n* = 8; Week 7–8, *n* = 10; Drug withdraw, *n* = 10). Tukey’s multiple comparisons test. (E) Locomotion of F0 monkeys in the novel cage. *n* = 5 for each monkey. (F) Durations of exploration, self-clasp, and self-injurious behaviors in DISC1 mutant heterozygote monkeys. *n* = 10 for each monkey. (G) Representative activity tracks for mutant and WT monkeys in the novel cage. Each point indicated one recording of each monkey. Two-tailed Welch’s *t*-test. Data are presented as mean ± S.E.M.

Continuous 24-h home-cage monitoring revealed elevated stereotypic and self-injurious behaviors in *DISC1* mutant monkeys, including repetitive pacing and twirling as categorized by standard behavioral ethogram definitions [29]. Self-injurious behavior mainly consisted of non-wounding skin contact with teeth. In F0 male mutants (46 M, 12-h trials), stereotypic behavior duration was significantly prolonged, along with increased self-injurious behavior during the daytime (Fig. 1A, Table S5). Notably, M3 displayed frequent self-biting—an atypical behavior for this species (Fig. S2A), while M4 (female) exhibited abnormal self-biting in the novel cage test (Fig. S2G–I). M2 and M3 showed marked increases in both frequency and duration of stereotypic behaviors (Fig. S2B and C). For F1 heterozygotes (30-min trials), home-cage recordings captured aberrant self-injurious and self-clasping behaviors (Fig. 1B, Table S6). These monkeys also spent less time hanging, suggesting heightened anxiety (Fig. 1B, Fig. S2D–F).

Following behavioral assessments, M3 was administered with 20 mg fluoxetine daily for two months, which is widely used in the treatment of depression, anxiety, and cognitive dysfunction [30]. Prior to treatment, M3 exhibited significantly more self-injurious and stereotypic behaviors compared to WT controls. Self-injurious behavior was partially alleviated (∼70% reduction vs. baseline) within the first two weeks of fluoxetine treatment, with effects lasting at least 1-month post-treatment (Fig. 1C). Stereotypic behavior showed ∼60% reduction after four weeks, and by six weeks, M3’s behavior was indistinguishable from WT baseline (∼80% reduction). However, one month after fluoxetine discontinuation, stereotypic behavior relapsed to pre-treatment levels (Fig. 1D). These findings align with fluoxetine’s documented effects in rhesus monkey models of obsessive-compulsive disorder [31] and self-injury [32].

Locomotion in novel environments and the home cage is a well-established metric in macaque behavioral studies [17–19]. At 46 months (46 M), mutant monkeys M1 and M3 displayed reduced exploratory behavior (Fig. 1E). In the human intruder test [28], *DISC1* heterozygotes (HET1 and HET2, 18 M) spent less time exploring compared to WT monkeys (Fig. 1F). Notably, HET2 engaged in persistent self-clasping at the cage edge throughout the test, while HET1 exhibited reduced movement and increased time hanging away from the intruder—unlike WTs, which typically approached the intruder (Fig. S3A–D). AI-based movement tracking indicated that M1, M3, and HET1 exhibited prolonged periods of corner-hanging behavior, a contrast to the WT monkeys who spent 75% of the 20-min observation period walking on the ground (Fig. 1G). This behavior is consistent with elevated stress or anxiety levels [33,34], further supporting the observed effects of *DISC1* disruption on aberrant exploratory and stress-related responses.

Sleep disturbances frequently co-occur with major neurological illness and have been reported in DISC1 mouse models [35]. To assess sleep patterns in the mutant monkey cohort, we monitored activity using actigraphy devices embedded in their collars. No significant differences were observed between WT and DISC1-mutant groups regarding total day/night time activity or their ratio (Fig. S4A and B). However, analysis of 5-min activity intervals revealed reduced daytime activity in the mutant monkeys, accompanied by brief naps or cage crawling as evidenced by video recordings (Fig. S4C). Nighttime video analysis categorized sleep into awake, transitional, and relaxed phases. Compared to age-matched controls, *DISC1*-mutant monkeys exhibited increased duration and frequency of transitional sleep, alongside decreased duration and reduced frequency of relaxed sleep (Fig. S4D). A similar sleep disturbance pattern was observed in 12 M heterozygote HET1, but not in HET2 (Fig. S4E). These data indicate fragmented sleep and reduced sleep efficiency in *DISC1*-mutant monkeys, with incomplete penetrance among F1 heterozygotes (HET1 affected, HET2 unaffected). This phenotype may emerge early in life and persist into adolescence in susceptible individuals. The above behavioral abnormalities of F0 and F1 mutant macaques were summarized in Table S7.

### Impaired social interaction

**Figure 2.**
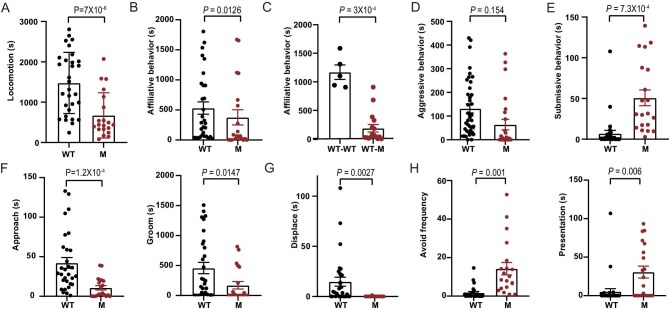
Impaired social behavior of *DISC1*-mutant macaques. Paired social interaction test of 1 h video recording. (A) Quantification for locomotion. (B–E) Quantification for social interaction behaviors including affiliative behaviors to the other (B), WT1’s affiliative behaviors toward the other (C), aggression (D) and submission (E). (F and G) Quantification for subtypes of affiliative behaviors (approach, groom, and displace). (H) Quantification for specific submissive behaviors (avoid and presentation). WT, *n* = 30; M, *n* = 20; WT–WT, *n* = 5; WT-M; *n* = 20. Two-tailed Welch’s *t*-test. Data are presented as mean ± S.E.M.

To assess social interaction in *DISC1*-mutant monkeys, we compared WT groups (three WT monkeys paired into two groups) with mutant (M) groups (one WT monkey paired with one mutant monkey). At 30 M, social interaction time did not differ between the two groups (Fig. S5A). *DISC1*-mutant monkeys also exhibited less movement, consistent with their behavior in the novel cage tests (Fig. 2A). Detailed behavioral analysis at 46 M revealed that mutants displayed decreased affiliative behaviors, along with increased submissive behaviors (Fig. 2B–E, Fig. S5B and C).

While submissive behavior increased overall, responses were heterogeneous across mutants: M1 and M2 were largely passive, M3 escalated to biting/fighting after being ignored by its WT partner, and M4 showed overt aggression (Fig. S5F). Across all subtypes of social behavior, mutant macaques showed significant differences in approaching, grooming, displacing, avoiding, and submissive presentation compared to WT controls (Fig. 2F–H, Fig. S5E). M2 and M3 exhibited prolonged lip-smacking behavior [36] Notably, mutants displayed fewer approaches and more avoidance, indicating reluctance to initiate or accept social interactions—mirroring negative symptoms (low motivation, social withdrawal) seen in neuropsychiatric disorders. In contrast, M4 displayed overt aggression, chasing and pulling the fur of WT monkeys (Fig. S5D). Analysis of the WT probe monkey (WT1, which interacted with both WT and mutant groups) revealed reduced affiliative behaviors toward mutants compared to its interactions with other WT partners (Fig. 2C).

Together, these results demonstrate distinct behavioral phenotypes in *DISC1*-mutant macaques. The three male mutants (M1, M2, and M3) exhibited reduced social interaction and increased avoidance, mirroring the negative symptoms (e.g. social withdrawal, low motivation) observed in neuropsychiatric disorders such as SZ, MDD, and ASD. These results suggest that *DISC1* disruption contributes to divergent behavioral dysregulation, recapitulating key aspects of human psychiatric pathology.

### Perceptual processing abnormality

Perceptual abnormality is a hallmark of cognitive impairment in SZ. The auditory oddball paradigm is used to assess sensory gating mechanisms for filtering irrelevant stimuli [37]. We recorded electroencephalographic (EEG) signals in anesthetized macaques during tone presentation. The auditory evoked potentials (AEPs) localized to frontal cortical regions were correlated with tone intensity (85 dB deviant vs. 60 dB standard) (Fig. S6A and B). Both groups exhibited characteristic event-related potentials (ERPs) with intensity-dependent P50 and N100 amplitudes (Fig. S6C and E). M3 displayed significantly increased modulation gain for P50 and N100 components across stimuli (Fig. S6D and F). Mismatch negativity (MMN), derived from deviant-standard AEP differences, demonstrated elevated peak amplitudes (100–250 ms post-stimulus) in M3 vs. WT controls (Fig. S6G and H). These results indicate aberrant sensory gating in *DISC1* mutants, characterized by hyper-responsivity to irrelevant auditory stimuli and impaired neural adaptation, with heightened MMN and distorted oscillatory activity. The enhanced AEP observed in M3 was recently reported in monkeys by drug injection (PCP/ketamine), which is believed to disrupt glutamatergic neurotransmission and cause SZ-like symptoms [38].

### Multisensory perceptual abnormality

**Figure 3.**
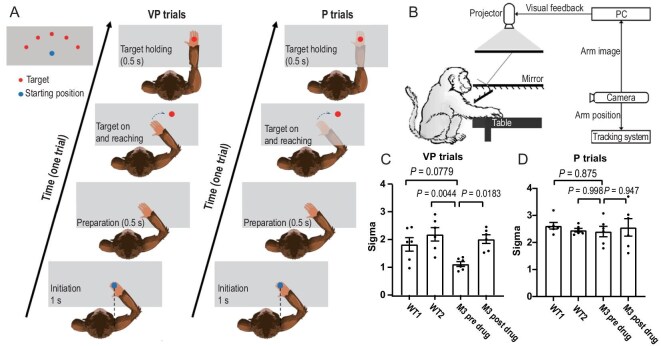
*DISC1*-mutant leads to multisensory perception abnormality. (A) Overview of the visual-proprioceptive (VP) and proprioceptive (P) task. The monkeys were instructed to hold their hands over the starting point (bottom middle dot) to initiate a trial. After a short preparation period, a virtual red dot was presented, and monkeys were required to place their P arm on the target and hold to get a juice reward. (B) The virtual-reality experimental system. (C) The sensory precision in VP task (*n* = 6). (D) The sensory precision in P task (*n* = 6). One-way analysis of variance (ANOVA). Data are presented as mean ± S.E.M.

To assess the impact of the DISC1 mutation on multisensory perception, we utilized a visual-proprioceptive multisensory-integration paradigm [39]. Using a virtual reality system, monkeys performed a touch-visual target task with or without a virtual arm moving synchronously with the real visual signal (Fig. 3B). Following placement of their hand at the starting point (Fig. 3A, blue dot) to initiate a trial, the visual signal of the arm was preserved in visual-proprioceptive (VP) task and hidden in the proprioceptive (P) task. Monkeys received a juice reward for successfully touching the target point (Fig. 3A, red dot). For each trial, we recorded the angular deviation of the real middle finger relative to the target. The standard deviation across 20 consecutive trials served as the sensory-precision index for each condition. Due to arm-length constraints, testing was limited to M3, WT1, and WT2.

**Figure 4.**
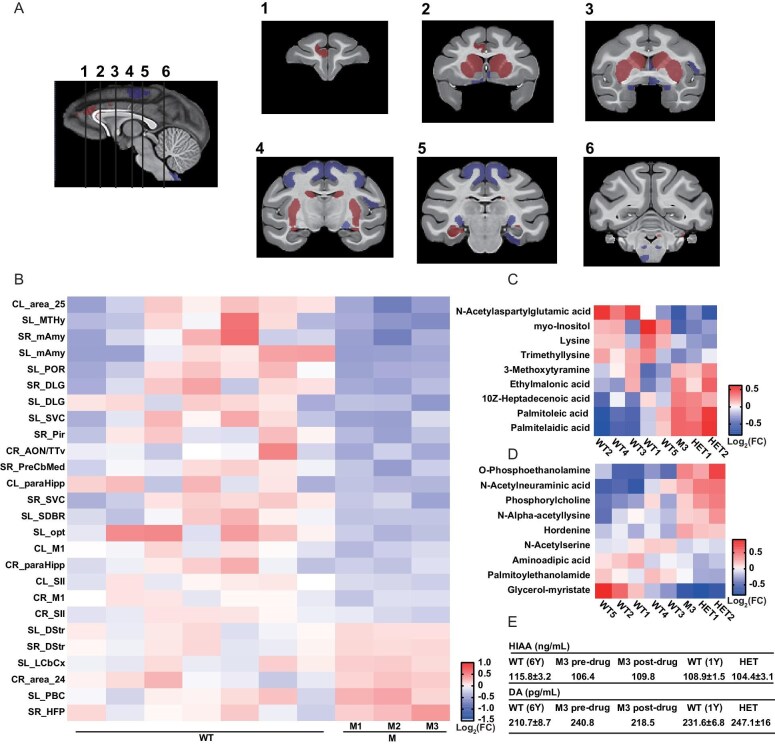
The brain structure and neuroendocrine metabolites change in DISC1-mutant monkeys. (A) Significant larger and smaller brain areas labeled on NMT2.1. with sagittal planes showing 6 coronal plane slices picked as demonstration of mainly changed areas. (B) Heatmap showing relative brain area volume in all samples with significant difference (*P* < 0.05). (C) Heatmap of blood metabolites with significant difference in DISC1-mutant monkeys, color indicated log_2_(foldchange). (D) Heatmap of cerebrospinal fluid metabolites with significant difference in DISC1-mutant monkeys. (E) ELISA results of blood DA and HIAA. M3 pre drug means before fluoxetine treatment. M3 post drug means after 1-month fluoxetine treatment.

Compared with WT1 and WT2, M3 exhibited significantly higher sensory precision in the VP condition (Fig. 3C), whereas no group difference emerged in the P condition (Fig. 3D). This pattern indicates that M3 up-weighted visual information during visual-proprioceptive integration. After fluoxetine treatment, M3’s VP precision dropped to WT levels. Given that the pre- vs. post-drug change in visual precision paralleled the change in DA signal (Fig. 4E), we suggest a dopamine-dependent increase in visual precision maybe a key driver of the multisensory-integration deficit observed in DISC1-mutant monkeys [40] and that fluoxetine can normalize this process. These findings are limited in sample sizes and require further validation.

### Altered brain structure and neuroendocrine metabolites

To determine whether DISC1 mutation affects brain structures, we used 3.0T MRI scanner to analyze three DISC1-mutant macaques (5Y) and seven aged-matched WT controls. Using NIMH Macaque Template (NMT2.1) as the hierarchical atlas [41], we obtained the Atlas of the Rhesus Macaque (ARM) segments of our data (Fig. 4A and B). Enlarged bilateral dorsal striatum in mutants indicates elevated dopaminergic function. Furthermore, primary motor cortex (CL_M1, CR_M1) and secondary somatosensory cortex (CL_SII, CR_SII) on both sides of brain exhibited reduced volume in mutant monkeys, indicating motor and somatosensory defects. Other structural abnormalities in mutant monkeys included reduced bilateral medial amygdaloid nucleus (ME) and anterior amygdaloid area; reduced bilateral dorsal lateral geniculate in thalamus; reduced volume of regions Sp5C, CuGr, and MdRt in the medulla. These results indicate potential impairments in perception and emotion related hypothalamic-pituitary-adrenal (HPA) axis and serotonergic (5-HT) systems (Fig. S7A and B).

To investigate whether structural changes caused neurotransmitter dysfunction, we performed targeted metabolomics on blood and cerebrospinal fluid (CSF) from *DISC1*-mutant monkeys (M3 at 75 M; HET1 and HET2 at 20 M) and age-matched WT controls (Fig. S8A–D). Blood metabolomics revealed decreased levels of N-acetylaspartylglutamic acid (NAAG, a glutamate/GABA modulator) and myo-inositol (a neurometabolite as a marker of glial cell activity), alongside elevated 3-methoxytyramine (a dopamine metabolite) (Fig. 4C). Indeed, myo-inositol has been reported to be reduced in CSF in patients with MDDs and affective disorders [42]. In addition, CSF metabolomics revealed increased level of hordenine, which has been identified as a DRD2 agonist recently [43], as well as membrane lipid metabolites phosphorylcholine and O-Phosphoethanolamine (Fig. 4D). The term Alanine, aspartate and glutamate metabolism was in the top 5 enriched KEGG pathways in both CSF and blood serum (Fig. S8E and F). ELISA-based analysis of plasma neurotransmitter metabolites demonstrated consistent abnormalities across mutant monkeys (M3, HET1, and HET2) including the tendency of elevated dopamine (DA) and reduced 5-hydroxyindoleacetic acid (5-HIAA, a serotonin metabolite) (Fig. 4E). Notably, fluoxetine treatment in M3 partly normalized these imbalances, reducing DA and increasing 5-HIAA and serotonin, consistent with the observed improvement in self-injurious and stereotypic behaviors (Figs 1C and D, 3C, Fig. S8G). These results align with structural alterations in dopaminergic and serotonergic pathways, supporting the hypothesis of neurotransmitter dysregulation underlying behavioral phenotypes. The therapeutic effects of fluoxetine further support the translational relevance of this model, demonstrating that pharmacological modulation of 5-HT can ameliorate DA/5-HT imbalances and associated behaviors, mirroring clinical outcomes in neuropsychiatric disorders.

### Excitatory and inhibitory neuron imbalance and synaptic dysfunction in snRNA-seq

**Figure 5:**
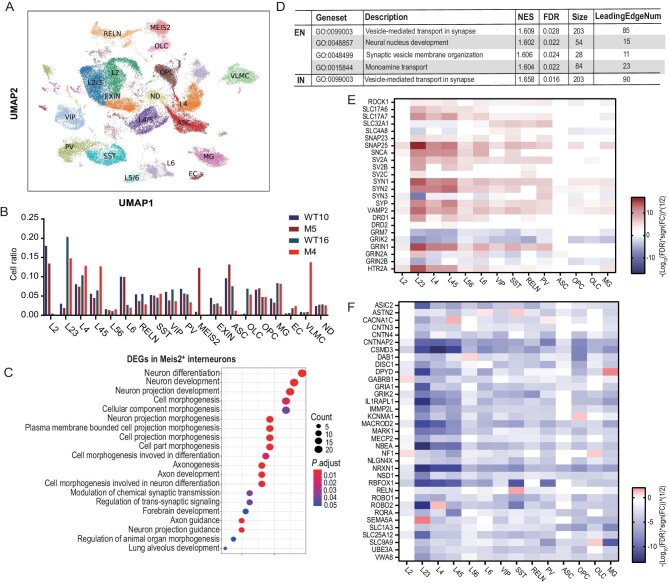
The sn-RNAseq results of dlPFC area from WT and *DISC1*-mutant monkey brains. (A) Uniform manifold approximation and projection (UMAP) plots depicting 44 758 cells from two mutant and two WT monkeys into 19 main cell types. Excitatory (L2, L2/3, L4, L4/5, L5/6, L6, and EXIN) and inhibitory neurons (PV, RELN, SST, VIP, and MEIS2) are divided into subtypes. (B) The ratios of indicated cell types in WT and mutant macaques. (C) GO enrichment of DEG in Meis2 ^+^ interneurons. (D) Significant GO enriched terms about synapses and neurons in WT16 and M4 excitatory neurons and inhibitory neurons. (E) Heatmap of synaptic genes and neurotransmitter receptor subunits (M4 vs. WT16) in main cell types. (F) Heatmap of autism risk genes (M4 vs. WT16) in main cell types.

To assess cell-type-specific involvement in *DISC1*-mutant macaques at early stages, we performed single nuclei RNA sequencing from the dorsolateral prefrontal cortex (dlPFC) of male fetal monkeys (WT10 and M5 at E130-E142) and adolescent female monkeys (WT16 and M4 at 3 years post-birth). Using well-established cell-type markers, we identified 19 neuronal and non-neuronal subtypes, consistent with previous reports in macaques (Fig. 5A, Fig. S9A and D) [44]. In both fetal and adolescent groups, we observed a decrease in L2, L2/3, reelin positive (RELN^+^), vasoactive intestinal polypeptide positive (VIP^+^), and parvalbumin positive (PV^+^) neurons (Fig. 5B, Fig. S9B). Notably, the fetal group exhibited an aberrant increase in *MEIS2* ^+^ interneurons, which was not seen in adolescents. Trajectory reconstruction revealed that interneurons in *DISC1*-mutant macaques preferentially differentiated toward the *MEIS2* ^+^ subtype (Fig. S9C). Gene ontology (GO) analysis of differentially expressed genes (DEGs) in *MEIS2 ^+^* inhibitory neurons highlighted ‘neuron differentiation’ as the top enriched pathway (Fig. 5C). The adolescent female group showed almost no *MEIS2* ^+^ interneurons (∼10 cells total in WT16 and M4), consistent with prior findings that Meis2 promotes GABAergic projection neuron generation primarily during embryogenesis [45]. This result suggests that *DISC1* mutation alters embryonic GABAergic neuron fate determination. In contrast, adolescent mutants displayed an increase in L4 and L4/5 excitatory neurons, alongside reduced astrocytes and expanded vascular leptomeningeal cells (Fig. 5B), indicating persistent dysregulation of cortical circuitry and neurovascular interactions.

The analysis of DEGs in excitatory neurons and interneurons revealed significant synaptic dysregulation in *DISC1*-mutant macaques (Fig. 5D). Adolescent female mutants exhibited increased expression of presynaptic protein mRNAs (*SYN1, SYN2, SV2C*, and *VAMP2*) in excitatory neurons, mirroring findings from human iPSC studies [8].

We also observed elevated expression of DISC1*-*regulated genes *RAC1* and *PDE4B* in these neurons. Notably, several neurotransmitter receptor subunits showed differential expression, including upregulation of *HTR2A* (5-*HT2A* serotonin receptor) that mediates psychedelic drug effects, and *DRD1* (dopamine receptor D1) which has been associated with ketamine-induced changes in mouse models [46] (Fig. 5E, Fig. S9E). These molecular alterations may underlie the observed neurochemical imbalances characterized by elevated dopamine and reduced serotonin levels in blood samples. GSEA analysis using the DisGeNET database further identified a downregulated autism-related gene module (Fig. 5F, Fig. S9F) across multiple cell types in the adolescent female mutant, suggesting potential convergence with neurodevelopmental disorder pathways.

## DISCUSSION

NHP models offer critical advantages over rodents for studying psychiatric disorders due to their neuroanatomical and behavioral complexity, particularly in domains such as social cognition and affective processing that are difficult to recapitulate in lower species. While spontaneous NHP models of conditions like depression and autism occur at low frequencies (2%–5% in captive populations) [31,47,48], recent advances in genetic engineering have enabled the development of targeted NHP models carrying mutations in genes such as *SHANK3* [20], *MECP2* [17,21], and *BMAL1* [18] that are implicated in neuropsychiatric diseases. In this study, we generated *DISC1* mutant macaques carrying a 5-bp deletion in exon 9, including mosaic males and heterozygous females. These animals exhibited a constellation of behavioral abnormalities with direct relevance to human psychopathology. Specifically, they displayed reduced exploratory behavior suggestive of motivational deficits, increased repetitive and self-injurious behaviors not typically observed in rodent *DISC1* models (a phenomenon that is naturally occurring in 5%–15% of WT macaques [49]), and marked social withdrawal mirroring symptoms observed in depression and anxiety disorders. Male mosaics and one heterozygous female demonstrated fragmented sleep characterized by reduced duration of relaxed sleep and increased state transitions, while another heterozygous female maintained normal sleep patterns. One mosaic male exhibited heightened visual precision and aberrant multisensory perceptual integration. These behaviors parallel the reduction in motivation and social interaction and increased anxiety in patients with MDD and anxiety disorders as well as perceptual disturbance. Self-harm and fragmented sleep are not reported in *DISC1* mutant rodent models. Also, expressed social behaviors are more complex and offer better validty in the *DISC1* mutant macaque compared to rodent models. Although we reported these behavioral phenotypes related to psychiatric disease, a key limitation of the present monkey model is its constrained ability to recapitulate core SZ phenotypes at the syndromic level. In particular, positive symptoms such as hallucinations and delusions are intrinsically subjective and language-dependent; because nonhuman primates cannot provide verbal reports of perceptual experiences or belief content, these domains cannot be directly validated and are at best inferred from indirect behavioral proxies with limited specificity. In addition, although nonhuman primates enable rigorous assessment of certain cognitive operations, the cognitive tasks typically used in monkeys are necessarily simplified, highly trained, and narrowly operationalized, and therefore may not fully capture cognitive deficits observed in patients with SZ.

Neurological disorders are widely recognized as brain disorders characterized by neurotransmitter imbalances and disruptions in region-specific neural circuits that govern behavior, cognition, and emotion. In our *DISC1*-mutant macaque model, neuroanatomical and neurochemical analyses revealed pathological changes with direct relevance to human psychiatric conditions. Specifically, we observed bilateral enlargement of the dorsal striatum accompanied by elevated serum dopamine levels—a neuropathological profile that mirrors findings in SZ and schizotypal personality disorder patients, where increased dopaminergic dendritic arborization has been proposed as one potential mechanism underlying striatal volume expansion [50]. Dorsal striatal enlargement accompanied by elevated CSF dopamine likely reflects a primary neurodevelopmental alteration of corticostriatal–dopamine systems, modeling a mechanistically relevant dimension of psychosis vulnerability. Concurrently, volumetric reductions were detected in the bilateral medial amygdaloid nucleus and anterior amygdaloid area, regions known to mediate aversive processing and consistently reported as structurally compromised in MDD [51]. The sole adult *DISC1*-mutant macaque, M3, underwent visual-proprioceptive integration testing [39] and displayed heightened visual acuity which were reversed by fluoxetine. These effects may respectively track the drug-associated shifts in dopaminergic tone [40]. Combined with the observation of decreased level of NAAG, myo-inositol, HIAA and increased level of 3-Methoxytyramine, our *DISC1*-mutant macaques exhibit a comorbid condition encompassing features of both SZ and depression, representing a more clinically relevant phenotype than that observed in existing DISC1 rodent models.

Emerging evidence implicates synaptic dysregulation and disrupted E/I balance as fundamental neurodevelopmental mechanisms underlying various mental disorders [2]. Our snRNA analysis of *DISC1*-mutant macaques revealed a significant reduction in parvalbumin-positive (PV^+^) interneurons, a critical GABAergic subpopulation whose depletion has been consistently documented in both postmortem studies of SZ and depression patients as well as in DISC1 rodent models [52]. It is considered that weakening inhibition and cortical gating contribute to motivational/social deficits, anxiety-like withdrawal, sleep fragmentation, and sensory/perceptual dysregulation. Notably, NMDA receptor antagonism (e.g. ketamine) has been shown to reduce the responses of putative PV^+^ fast-spiking interneurons in primate lateral prefrontal cortex and to induce selective working-memory deficits supporting a link between PV-interneuron dysfunction and SZ-relevant cognitive phenotypes [53]. Moreover, large-scale single-nucleus and spatial transcriptomic profiling of dorsolateral prefrontal cortex in SZ reported preferential vulnerability of upper cortical layers, including reduced abundance and marked molecular alterations in upper-layer GABAergic interneuron subclasses encompassing PV/VIP-related types [54]. Transcriptomic profiling further demonstrated widespread disturbances in synaptic function, particularly in vesicle-mediated transport pathways, consistent with previous reports of impaired presynaptic neurotransmitter release in iPSC-derived forebrain neurons carrying DISC1 mutations [8]. In adolescents, mutants exhibited upregulation of presynaptic genes (*SYN1/2, SV2C*, and *VAMP2*), suggesting heightened excitatory synaptic drive, and this together with reduced inhibitory interneurons, could exacerbate E/I imbalance and promote repetitive/self-injurious behaviors. Excitatory neurons also showed increased expression of DRD1 and HTR2A, along with DISC1-linked signaling genes (*RAC1* and *PDE4B*), indicating altered dopamine/serotonin responsiveness and synaptic signaling that may relate to elevated dopamine and reduced serotonin.

A large number of autism-risk genes had lower expression level in female adolescent DISC1 mutant macaque compared with wild type control, including two well-studied monogenic ASD genes *NRXN1* and *MECP2* [17,55], suggesting our *DISC1*-mutant macaque model captures conserved neurodevelopmental pathology across psychiatric conditions. The availability of NHP brain tissue is inherently constrained, and in our case the number of age- and sex-matched animals—particularly WT controls—was very small, precluding additional biological replicates. As a result, statistical power is reduced and some observed differences may be sensitive to individual variability.

The *DISC1*-mutant macaques exhibited substantial phenotypic heterogeneity across both the F0 founder generation and the F1 heterozygous generation. Behavioral outcomes were variable in both onset and trajectory. For example, male mutants (M1–M3) showed no significant behavioral differences at 30 months, but by 46 months they displayed divergent locomotor activity and social interaction patterns. In females, one F0 animal (M4) developed early-onset stereotypic and self-injurious behaviors by 30 months. Notably, M3 was the only animal that survived long-term and showed behavioral improvement after a 2-month fluoxetine treatment, with reduced stereotypy and self-injury. Strikingly, behavioral abnormalities appeared earlier in the F1 generation, as two heterozygous female mutants exhibited stereotypic and self-injurious behaviors as early as 18 months. Sleep disturbance was relatively consistent among F0 animals but more variable in F1. This phenotypic heterogeneity is consistent with observations in human DISC1 disruption. In the Scottish pedigree carrying the balanced translocation t(1;11) (q43; q21), which disrupts *DISC1*, carriers present with a wide range of major psychiatric disorders, including SZ, MDD, generalized anxiety disorder, schizoaffective disorder, and alcoholism [7]. Disruption of DISC1 can lead to diverse, developmentally dynamic behavioral phenotypes, which explains the phenotypic heterogeneity observed in our DISC1-mutant macaques.

Due to the low survival rate of DISC1-mutant monkeys, our findings are based on limited sample size which requires validation in larger cohorts. The onset and progression of symptoms showed generational differences (F1 generation showed abnormalities earlier than F0), which may be partly affected by gender differences (M4 showed abnormal aggressive behaviors earlier than male F0 WT macaques, while F1 generations are female). Future investigations should employ longitudinal multimodal assessments in F1-generation mutants, including continuous behavioral recording, neuroendocrine profiling, and structural/functional neuroimaging, to deeply understand how DISC1 mutations disrupt neurodevelopmental trajectories. The generational differences also warrant further investigation to determine whether they reflect complex gene-environment interactions or transgenerational epigenetic effects.

## MATERIALS AND METHODS

In this research, we used cynomolgus monkeys as model animals. We strictly followed the guidelines of the Institutional Animal Care and Use Committee and approved experimental procedures (IACUC numbers: 20240623001, ION-20180105 and ION-2019043). The monkeys were housed in cages with appropriate volume, allowing monkeys to move freely, in the environment with 22–25°C temperature, ∼50% humidity, and a regular 12-h light-dark cycle. In the first 30 months after birth, the physical examination was taken monthly to record weight, abdomen circumference, body length, head circumference, heart rate, breath rate, and body temperature.

More detailed materials and methods are available in the Supplementary data.

## Supplementary Material

nwag200_Supplemental_Files

## Data Availability

The single-cell RNA-seqencing data generated in this study have been deposited in the BioBigData Database Center (BMDC) of the National Genomics Data Center (NGDC), China National Center for Bioinformation (CNCB) under the project accession number OEP00006559. The data are publicly accessible at https://www.biosino.org/node/project/detail/OEP00006559. The authors declare that all other data supporting the findings of this study are available within the paper and its supplementary information files.
