# Supplementary material for: DISC1 mutant macaques capture behavioral and neural hallmarks of psychiatric disease
Source: Natl Sci Rev. 2026 Mar 28;13(8):nwag200. doi: 10.1093/nsr/nwag200 (PMC13166873; doi:10.1093/nsr/nwag200)
Supplement: nwag200_Supplemental_Files [file nwag200_supplemental_files.zip › Supplementary files.doc]

**SUPPLEMENTARY INFORMATION**

**Fig. S1 | Summary of DISC1 mutations in F0 and F1 macaques. A,** sgRNA information used to establish the DISC1 mutated monkeys. 8 embryos were injected to test the efficiency of the edition for each sgRNA. **B**, Information for the embryos injected and survival situation of the macaques. **C**, Genotyping of 10 tested embryos with single sperm injection from M3. The ratio of sperms with the mutation is similar to skin tissues. **D**, Pie charts of genotype (indels) from ear tip tissue of live mutant macaques. **E,** Results of genotyping from cortex, cerebellum, muscle, liver, and skin of aborted F0 macaques, the ratio indicated the edited alleles out of the total. **F**, Results of TA-clone genotyping from skin of F1 macaques**. G**, DNA gel electrophoresis verification for the presence of +5bp indel using primers containing the inserted sequence. **H,** Western-blot results of DISC1 protein levels in samples of macaques’ prefrontal cortex using β-actin as loading control. **I**, Body weight of F0 (45M) and F1 (12M) DISC1-mutant macaques and WT control. **J**, Body weight, crown-rump length and head circumference of F0 DISC1-mutant macaques (M1, M2, M3, M4) compared with age and sex matched WT macaques (WT1, WT2, WT3) in the first 30 months. Data are presented as mean ± S.D. Two-tailed Welch’s t-test.

**Fig. S2 | DISC1 mutant monkeys display increased self-injurious and stereotypic behavior in the home cage. (A-C),** Time spent on self-injurious and stereotypic behavior of each F0 DISC1-mutant monkeys and WT controls. n=6 trails for each monkey. **(D-F)**, Time spent on self-injurious, self-clasp and hanging behavior of each F1 DISC1 heterozygous (HET) monkeys and WT controls. WT13, n=10; WT14, n=11; WT15, n=12; HET1, n=13; HET2, n=11. **G**, M4 exhibited higher frequency of self-injurious behavior in the novel environment. WT, n=15; M4, n=5. **H**, Representative image of the wound on M3 caused by self-bite one year after fluoxetine withdraw. **I**, Representative images of self-injurious behavior of mutant macaques (M4, M3, HET1). Two-tailed Welch’s t-test. Data are presented as mean ± S.E.M.

**Fig. S3 |F1 DISC1-mutant monkeys exhibited shorter time on exploration in human intruder test. A-B**, Analysis of human intruder test containing 2 control sessions (12-min each), one human standing with no eye contact session (profile, 4-min) and one human staring the monkey session (stare, 4-min). Data shown are quantifications for the time of movement, hanging, staying at the corner, and self clasp when human intruder standing in front of the cage not looking at the monkey. (A) or under the circumstance of human staring at the monkey (B). n=5 trails for each monkey. **C,** Movement duration when human staring (stare) or avoiding eye contact (profile) with the monkey. WT, n=15; HET, n=10. **D**, Representative image for DISC1 heterozygote monkeys with human intruder at the left side of the cage. Two-tailed Welch’s t-test. Data are presented as mean ± S.E.M.

**Fig. S4 | Altered sleep of DISC1-mutant macaques. A**, Continuous 7-day locomotor activity record of DISC1-mutant and WT monkeys at 46M. **B**, Summary of physical activity at day, at night and the day/night ratio. **C**, The 24-hr distribution of averaged physical activity of DISC1-mutant and WT monkeys. Black lines on the top indicate time points with significant differences (P < 0.05), and SEMs are shown as shaded areas. WT, n=13; M, n=20. **D-E**, Summary of duration and frequency of awake, transitional sleep and relaxed sleep in F0 DISC1-mutant (D) (WT, n=8; M n=12) and F1 DISC1- HET and age-matched WT controls (E) (WT, n=14; HET1, n=5; HET2, n=5). Two-tailed Welch’s t-test. Each point is one recording. Data are presented as mean ± S.D.

**Fig. S5 | Impaired social behavior of DISC1-mutant monkeys at 46M. A**, Paired social interaction test of 1-hr video recording at 30M. WT-WT means the WT monkey acts towards the other WT monkey. WT-M means the WT monkey act towards mutant monkeys. n=13 for each group. Data are presented as mean ± S.D. **B-F**, Quantification for social interaction behavior including approach (b), submission (C), and aggressive behaviors (grab, chase)(D). (E, F) Quantification for submissive behaviors (presentation, avoid) and lipsmack. WT1, n=20; WT2, n=10; M1, n=5; M2, n=5; M3, n=10. Each point is one recording. Two-tailed Welch’s t-test. Data are presented as mean ± S.E.M.

**Fig. S6 | AEP were enhanced in the DISC1-mutant macaque. A,** The configuration of EEG cap used in the recording. **B**, The probability of regular tone (60 dB) was 20% and that of irregular tone (85 dB) was 80% in a randomized sequence. **C-F**, Comparison of AEP for 60 dB-tone and 85 dB-tone in WT and mutant macaques. **G-H**, Comparison OF MMN in WT and mutant macaques. The black lines in (F) and (H) mark the parts with significant difference. P<0.05, two-tailed Welch’s t-test. Data are presented as mean ± S.E.M.

**Fig. S7 | The brain structure change (ARM level 6) in DISC1-mutant monkeys. A**, Significant larger (red) and smaller (blue) brain areas labeled on NMT2.1. Sagittal planes showing 6 coronal plane slices picked for the demonstration of mainly changed areas. **B**, heatmap showing the relative brain area volume in all samples.

**Fig. S8 | Metabolomics analysis of CSF and blood samples from DISC1-mutant monkeys. A**, Volcano plot of metabolomics in CSF from DISC1-mutant monkeys and wild-type controls. **B**, Log2 (FC) of metabolomics in CSF with significant difference. **C**, Bubble chart of KEGG enrichment of metabolomics in CSF with significant differences. **D**, Volcano plot of metabolomics in blood from *DISC1*-mutant monkeys and WT controls. **E**, Log2 (FC) of metabolomics in blood with significant difference. **F**, Bubble chart of KEGG enrichment of metabolomics in blood with significant difference. **G,** Concentrations of glutamine, serotonin, acetylcholine, trans-aconitic acid, pyruvic acid, isocitric acid concentration in the CSF from indicated monkeys before and after fluoxetine treatment for 30 days.

**Fig. S9 | snRNAseq results of dlPFC area from WT and DISC1-mutant monkey brains. A**, Uniform manifold approximation and projection (UMAP) plots depicting 44758 cells from 2 mutant and 2 WT monkeys individually into 19 main cell types. Excitatory (L2, L2/3, L4, L4/5, L5/6, L6, EXIN) and inhibitory neurons (PV, RELN, SST, VIP, MEIS2) are divided into subtypes. **B**, Marker gene expression level in UMAP of excitatory and inhibitory neurons. **C**, Reconstruction of interneuron developmental trajectory. In the DISC1-mutant, more interneurons are in the state in with high Meis2 expression (right corner). **D.** Cell-type annotation based on the expression of well-established marker genes. **E,** Heatmap of GO enrichment in GSEA analysis for main cell types. **F.** GSEA analysis showing the enrichment of mental disease related pathways in main cell types.

**Table. S1 |Summary of edited and WT macaques used in this study.** IM: incomplete mutant mosaic. CM: complete mosaic. HET: heterozygote with +5bp indel.

|  | Label | Sex | Genotype | Edit | Death age | tissue used |  |  |  |  | observation time |  |  |
| --- | --- | --- | --- | --- | --- | --- | --- | --- | --- | --- | --- | --- | --- |
| DISC1 | M1 | M | F0 | IM | 52M | cortex | cerebellum | muscle | liver | skin | 30M | 46M |  |
|  | M2 | M | F0 | CM | 71M | cortex |  |  |  | skin | 30M | 46M |  |
|  | M3 | M | F0 | IM | alive |  |  |  |  | skin | 30M | 46M | 70M |
|  | M4 | F | F0 | CM | 35M | cortex |  |  |  | skin | 30M |  |  |
|  | M5 | M | F0 | CM | E130 | cortex | cerebellum | muscle | liver | skin |  |  |  |
|  | M6 | M | F0 | CM | E130 | cortex | cerebellum | muscle | liver | skin |  |  |  |
|  | M7 | M | F0 | IM | E85 | cortex | cerebellum |  |  | skin |  |  |  |
|  | M8 | M | F0 | CM | D158 | cortex |  | muscle | liver | skin |  |  |  |
|  | M9 | M | F0 | CM | D163 | cortex | cerebellum |  |  | skin |  |  |  |
|  | M10 | M | F0 | IM | D145 | cortex | cerebellum | muscle |  | skin |  |  |  |
|  | M11 | F | F0 | IM | E85 | cortex | cerebellum | muscle |  | skin |  |  |  |
|  | M12 | F | F0 | CM | E151 | cortex |  | muscle | liver |  |  |  |  |
|  | M13 | F | F0 | CM | D157 | cortex | cerebellum |  |  | skin |  |  |  |
|  | M14 | F | F0 | CM | D151 | cortex | cerebellum |  |  | skin |  |  |  |
|  | HET1 | F | F1 | HET | alive |  |  |  |  | skin | 18M |  |  |
|  | HET2 | F | F1 | HET | alive |  |  |  |  | skin | 18M |  |  |
|  | HET3 | M | F1 | HET | E144/D8 | cortex |  |  |  | skin |  |  |  |
|  | HET4 | M | F1 | HET | E163/D27 | cortex |  |  |  | skin |  |  |  |
|  | HET5 | F | F1 | HET | E153/D16 | cortex |  |  |  | skin |  |  |  |
|  | HET6 | F | F1 | HET | E101 | cortex |  |  |  | skin |  |  |  |
| WT | WT1 | M |  | WT | alive |  |  |  |  | skin | 30M | 46M | 70M |
|  | WT2 | M |  | WT | alive |  |  |  |  | skin | 30M | 46M | 70M |
|  | WT3 | M |  | WT | alive |  |  |  |  | skin | 30M | 46M | 70M |
|  | WT4 | F |  | WT | alive |  |  |  |  | skin | 30M |  |  |
|  | WT5 | F |  | WT | alive |  |  |  |  | skin | 30M |  |  |
|  | WT6 | F |  | WT | alive |  |  |  |  | skin | 30M |  |  |
|  | WT7 | F |  | WT | E100 | cortex |  |  |  | skin |  |  |  |
|  | WT8 | F |  | WT | E140 | cortex |  |  |  | skin |  |  |  |
|  | WT9 | F |  | WT | E152 | cortex |  |  |  | skin |  |  |  |
|  | WT10 | M |  | WT | E142 | cortex |  |  |  | skin |  |  |  |
|  | WT11 | M |  | WT | E142/D5 | cortex |  |  |  | skin |  |  |  |
|  | WT12 | M |  | WT | E159 | cortex |  |  |  | skin |  |  |  |
|  | WT13 | F |  | WT | alive |  |  |  |  | skin | 18M |  |  |
|  | WT14 | F |  | WT | alive |  |  |  |  | skin | 18M |  |  |
|  | WT15 | F |  | WT | alive |  |  |  |  | skin | 18M |  |  |
|  | WT16 | F |  | WT | 36M | cortex |  |  |  | skin |  |  |  |

**Table. S2 | Off-target screening with fewer than 3 mismatches.** The result of searching sgRNA sequences using Cas-OFFinder. Potential off-target sites with 3 mismatches are listed with their sequence, chromosome, location, types, and gene names.

| #Bulge type | crRNA | DNA | Chromosome | Position | Direction | Mismatches | Bulge Size | type | gene |
| --- | --- | --- | --- | --- | --- | --- | --- | --- | --- |
| X | CATTAACATCAGAGAGAGAANGG | tATTAACAaCAGAGAGAtAAAGG | chr1 | 28830750 | - | 3 | 0 | exon | FMO2 |
| X | CATTAACATCAGAGAGAGAANGG | CATTgACtTCAGAGAGAGAcAGG | chr1 | 139234157 | + | 3 | 0 | intron | ALG14,TLCD4 |
| X | CATTAACATCAGAGAGAGAANGG | CATTtACcTgAGAGAGAGAATGG | chr1 | 186549905 | + | 3 | 0 |  | LOC123568732 |
| X | CATTAACATCAGAGAGAGAANGG | CATccACATCtGAGAGAGAATGG | chr1 | 188505214 | + | 3 | 0 | intron | TSPAN1 |
| X | GGGACTCCTCAGCAAGCTGNGG | GGGcCTCCaCAGCcAGCTGCAGG | chr1 | 95034892 | - | 3 | 0 |  |  |
| X | GGGACTCCTCAGCAAGCTGNGG | ttGACTCaTCAGCAAGCTGAAGG | chr1 | 203879482 | + | 3 | 0 |  |  |
| X | GGGACTCCTCAGCAAGCTGNGG | caGACTCaTCAGCAAGCTGATGG | chr1 | 223295869 | - | 3 | 0 | exon | DISP3 |
| X | CATTAACATCAGAGAGAGAANGG | CATTcACATCAGgGAGgGAAAGG | chr2 | 48034620 | - | 3 | 0 |  |  |
| X | CATTAACATCAGAGAGAGAANGG | CtTTAAgATCAGgGAGAGAAAGG | chr2 | 167034904 | - | 3 | 0 | intron | LSAMP |
| X | CATTAACATCAGAGAGAGAANGG | CATTgACATaAGtGAGAGAAAGG | chr2 | 191870036 | + | 3 | 0 |  |  |
| X | GGGACTCCTCAGCAAGCTGNGG | GGGtCaCCTCAGCAAGgTGTGGG | chr2 | 42631194 | - | 3 | 0 |  |  |
| X | GGGACTCCTCAGCAAGCTGNGG | tGGACTCCTCAGCAAtCTcTAGG | chr3 | 162768341 | - | 3 | 0 | intron | CPED1 |
| X | GGGACTCCTCAGCAAGCTGNGG | GGGgCTCCTCgGCAcGCTGCCGG | chr3 | 191195165 | - | 3 | 0 |  |  |
| X | CATTAACATCAGAGAGAGAANGG | CATTAACATCgGAGAGgcAATGG | chr4 | 15613922 | + | 3 | 0 |  |  |
| X | CATTAACATCAGAGAGAGAANGG | aATTAACAaCAGAaAGAGAAAGG | chr4 | 61539592 | + | 3 | 0 |  |  |
| X | GGGACTCCTCAGCAAGCTGNGG | GGaACTCCTgAGCAAaCTGTGGG | chr4 | 12932886 | - | 3 | 0 |  | LOC135970300,LOC123573063 |
| X | GGGACTCCTCAGCAAGCTGNGG | GcaACTCCTCAGCAAGCaGAAGG | chr4 | 34255713 | - | 3 | 0 |  |  |
| X | GGGACTCCTCAGCAAGCTGNGG | tGGACTCCTCAGCAAcCTcGTGG | chr4 | 41426763 | + | 3 | 0 |  |  |
| X | CATTAACATCAGAGAGAGAANGG | aATaAACATCAcAGAGAGAATGG | chr5 | 17477535 | + | 3 | 0 |  |  |
| X | GGGACTCCTCAGCAAGCTGNGG | GaGACTtCTCAGCAAGCTcAGGG | chr5 | 23174907 | + | 3 | 0 | intron | PPARGC1A |
| X | GGGACTCCTCAGCAAGCTGNGG | GGGgCTCCTCAaCAAcCTGAGGG | chr5 | 51644860 | - | 3 | 0 |  |  |
| X | CATTAACATCAGAGAGAGAANGG | CATcAACATCAtAcAGAGAAAGG | chr6 | 158690058 | - | 3 | 0 | intron | LOC141407091,GLRA1 |
| X | GGGACTCCTCAGCAAGCTGNGG | GGGACTCCcCtGaAAGCTGGTGG | chr6 | 5808510 | + | 3 | 0 | intron | ICE1 |
| X | GGGACTCCTCAGCAAGCTGNGG | GGGACTCCcCgGCAAGCTGCTGG | chr6 | 94237381 | + | 2 | 0 |  | LOC102140147 |
| X | GGGACTCCTCAGCAAGCTGNGG | GcGACatCTCAGCAAGCTGATGG | chr6 | 145226910 | - | 3 | 0 |  | LOC135917432 |
| X | GGGACTCCTCAGCAAGCTGNGG | tGGACTCCTCAGgAgGCTGTGGG | chr6 | 176596883 | + | 3 | 0 | intron | DOCK2 |
| X | GGGACTCCTCAGCAAGCTGNGG | GGGcCTCCTgAGCtAGCTGAAGG | chr6 | 177189152 | - | 3 | 0 | intron | KCNIP1,KCNMB1 |
| X | CATTAACATCAGAGAGAGAANGG | CATTAAaAagAGAGAGAGAATGG | chr7 | 99299665 | + | 3 | 0 | exon | GPR33 |
| X | CATTAACATCAGAGAGAGAANGG | CAcTAACtcCAGAGAGAGAATGG | chr8 | 69156873 | - | 3 | 0 |  |  |
| X | GGGACTCCTCAGCAAGCTGNGG | GGGcCTCaTCAGCAAcCTGCTGG | chr8 | 145971565 | - | 3 | 0 |  | LOC141407527 |
| X | CATTAACATCAGAGAGAGAANGG | CATTAgaAaCAGAGAGAGAATGG | chr9 | 50906596 | - | 3 | 0 |  | LOC107130780 |
| X | CATTAACATCAGAGAGAGAANGG | CAgTcACATtAGAGAGAGAAGGG | chr9 | 69309127 | + | 3 | 0 | intron | KCNMA1 |
| X | GGGACTCCTCAGCAAGCTGNGG | tGGgaTCCTCAGCAAGCTGGGGG | chr9 | 45455187 | + | 3 | 0 |  |  |
| X | GGGACTCCTCAGCAAGCTGNGG | GGGACTCCaCccCAAGCTGGAGG | chr10 | 31373625 | + | 3 | 0 |  |  |
| X | GGGACTCCTCAGCAAGCTGNGG | GGGACTCCaCAGCtcGCTGGTGG | chr10 | 66431799 | - | 3 | 0 |  |  |
| X | GGGACTCCTCAGCAAGCTGNGG | GGGACaCCTCAGCcAGCTcCAGG | chr10 | 80459537 | + | 3 | 0 |  |  |
| X | GGGACTCCTCAGCAAGCTGNGG | GGGcCTCCTCAGCAAGCctCTGG | chr10 | 85684461 | + | 3 | 0 |  | LOC102138723 |
| X | TACAGCTAAAGACCTCACCGNGG | aAaAGCTAAAGtCCTCACCGTGG | chr11 | 75172614 | + | 3 | 0 |  |  |
| X | GGGACTCCTCAGCAAGCTGNGG | GGGACgCaTCAtCAAGCTGGTGG | chr11 | 86530521 | + | 3 | 0 |  |  |
| X | GGGACTCCTCAGCAAGCTGNGG | GGGACTCCTtAcCAgGCTGATGG | chr11 | 97919653 | - | 3 | 0 |  |  |
| X | GGGACTCCTCAGCAAGCTGNGG | GGGACTCCTCAGttAcCTGCAGG | chr11 | 110351739 | - | 3 | 0 | intron | C11H12orf42 |
| X | TACAGCTAAAGACCTCACCGNGG | TACAGgTAAAGACCTCAgCcAGG | chr12 | 120605260 | + | 3 | 0 |  |  |
| X | GGGACTCCTCAGCAAGCTGNGG | GGaACTCCTCAGgAgGCTGGAGG | chr12 | 20320743 | - | 3 | 0 | intron | TMEM163 |
| X | GGGACTCCTCAGCAAGCTGNGG | GGGAgTCCTCAGgAAGCaGCAGG | chr12 | 95559833 | + | 3 | 0 | intron | TYW5 |
| X | GGGACTCCTCAGCAAGCTGNGG | GGGAgTCCTCAaCAAcCTGCTGG | chr13 | 18597794 | - | 3 | 0 |  |  |
| X | GGGACTCCTCAGCAAGCTGNGG | aGGAaTCCTCAGgAAGCTGTGGG | chr13 | 25476595 | - | 3 | 0 |  |  |
| X | GGGACTCCTCAGCAAGCTGNGG | GGGcaTCCTCAGCAAGtTGAAGG | chr13 | 37152205 | - | 3 | 0 | intron | SUCLG1 |
| X | GGGACTCCTCAGCAAGCTGNGG | GGGACTCaTCAGaAAGCTtTAGG | chr13 | 83771222 | + | 3 | 0 |  |  |
| X | TACAGCTAAAGACCTCACCGNGG | TACAGCTAAAGAtCTCtCCtTGG | chr14 | 42646523 | + | 3 | 0 |  | LOC102139027 |
| X | CATTAACATCAGAGAGAGAANGG | CATTAAaATCAGAaAaAGAAAGG | chr14 | 60063624 | - | 3 | 0 |  |  |
| X | CATTAACATCAGAGAGAGAANGG | CAaTAAtATCAGAGAcAGAAAGG | chr14 | 87421210 | + | 3 | 0 |  |  |
| X | CATTAACATCAGAGAGAGAANGG | CATTAACtTCAGAGgGAGAgGGG | chr14 | 115077399 | + | 3 | 0 |  |  |
| X | GGGACTCCTCAGCAAGCTGNGG | tGGACTCCTCAGaAAGaTGGGGG | chr14 | 12502349 | + | 3 | 0 | intron | SYT7 |
| X | GGGACTCCTCAGCAAGCTGNGG | GGaACTaCcCAGCAAGCTGTTGG | chr14 | 54733925 | - | 3 | 0 |  |  |
| X | GGGACTCCTCAGCAAGCTGNGG | GaGcCTCCTCAGtAAGCTGAGGG | chr14 | 116382823 | - | 3 | 0 | intron | DSCAML1 |
| X | CATTAACATCAGAGAGAGAANGG | CAaTAACtTCAGAGAcAGAAGGG | chr15 | 52009255 | + | 3 | 0 |  |  |
| X | CATTAACATCAGAGAGAGAANGG | CcTgAAaATCAGAGAGAGAAAGG | chr16 | 7243307 | + | 3 | 0 | intron | DLG4 |
| X | CATTAACATCAGAGAGAGAANGG | CtTTtACATCAGtGAGAGAAAGG | chr16 | 69265955 | + | 3 | 0 | intron | MARCHF10 |
| X | GGGACTCCTCAGCAAGCTGNGG | GGGcCTCCTCAGCAgGCTGGTGG | chr16 | 43376811 | - | 2 | 0 | intron | DDX52 |
| X | GGGACTCCTCAGCAAGCTGNGG | GGGcaTCCTCAGCAAGaTGCTGG | chr16 | 73494233 | - | 3 | 0 |  |  |
| X | CATTAACATCAGAGAGAGAANGG | tATcAACATCAGAGtGAGAAGGG | chr17 | 60404288 | - | 3 | 0 |  |  |
| X | GGGACTCCTCAGCAAGCTGNGG | GGGACTtCTCAaCAAGCTGTTGG | chr17 | 26573305 | - | 2 | 0 |  | LOC107127949 |
| X | GGGACTCCTCAGCAAGCTGNGG | GGGACaCCTCAGCAAaaTGATGG | chr17 | 48929439 | - | 3 | 0 |  |  |
| X | CATTAACATCAGAGAGAGAANGG | aATTAAaAgCAGAGAGAGAATGG | chr18 | 30788836 | - | 3 | 0 |  |  |
| X | CATTAACATCAGAGAGAGAANGG | CATTgACAaCAGAtAGAGAAAGG | chr18 | 45880004 | - | 3 | 0 |  | LOC102131594 |
| X | GGGACTCCTCAGCAAGCTGNGG | GGGcCaCCTCtGCAAGCTGAGGG | chr18 | 35366821 | + | 3 | 0 |  |  |
| X | GGGACTCCTCAGCAAGCTGNGG | GGGgCTCtTCAGCAAGCTGGTGG | chr18 | 49279691 | + | 2 | 0 |  |  |
| X | GGGACTCCTCAGCAAGCTGNGG | tGGAaaCCTCAGCAAGCTGAAGG | chr18 | 69662370 | + | 3 | 0 |  |  |
| X | CATTAACATCAGAGAGAGAANGG | aATTAACATCAGgtAGAGAAGGG | chr20 | 54201814 | + | 3 | 0 |  |  |
| X | GGGACTCCTCAGCAAGCTGNGG | GGGtCTCCTCAcCAtGCTGTGGG | chr20 | 29417992 | - | 3 | 0 |  |  |
| X | CATTAACATCAGAGAGAGAANGG | gAcTAACAgCAGAGAGAGAATGG | chrX | 54641353 | + | 3 | 0 |  | LOC102134461 |
| X | GGGACTCCTCAGCAAGCTGNGG | GGGACTgaTCAGCAAGaTGTGGG | chrX | 66582054 | + | 3 | 0 |  |  |
| X | GGGACTCCTCAGCAAGCTGNGG | GGGcCTCCaaAGCAAGCTGAAGG | chrX | 150788951 | + | 3 | 0 |  |  |

**Table. S3 | Summary of off-target sequencing.** Results of sanger sequencing of 19 potential off-target sites in exon or intron from 17 *DISC1* mutant macaques. (M1, M2, M3, M4, M5, M6, M7, M9, M10, M11, M13, M14, HET1, HET2, HET4, HET5, HET6). Only M6 detected a 5 bp insertion at intron of *ALG14/TLCD4*.

|  | crRNA | DNA | Chromosome | Position | Direction | Mismatches | type | gene | off-target detected |
| --- | --- | --- | --- | --- | --- | --- | --- | --- | --- |
| 1 | CATTAACATCAGAGAGAGAANGG | tATTAACAaCAGAGAGAtAAAGG | chr1 | 28830750 | - | 3 | exon | FMO2 |  |
| 2 | CATTAACATCAGAGAGAGAANGG | CATTgACtTCAGAGAGAGAcAGG | chr1 | 139234157 | + | 3 | intron | ALG14,TLCD4 | M6 +5bp |
| 3 | CATTAACATCAGAGAGAGAANGG | CATccACATCtGAGAGAGAATGG | chr1 | 188505214 | + | 3 | intron | TSPAN1 |  |
| 4 | GGGACTCCTCAGCAAGCTGNGG | caGACTCaTCAGCAAGCTGATGG | chr1 | 223295869 | - | 3 | exon | DISP3 |  |
| 5 | CATTAACATCAGAGAGAGAANGG | CtTTAAgATCAGgGAGAGAAAGG | chr2 | 167034904 | - | 3 | intron | LSAMP |  |
| 6 | GGGACTCCTCAGCAAGCTGNGG | tGGACTCCTCAGCAAtCTcTAGG | chr3 | 162768341 | - | 3 | intron | CPED1 |  |
| 7 | GGGACTCCTCAGCAAGCTGNGG | GaGACTtCTCAGCAAGCTcAGGG | chr5 | 23174907 | + | 3 | intron | PPARGC1A |  |
| 8 | CATTAACATCAGAGAGAGAANGG | CATcAACATCAtAcAGAGAAAGG | chr6 | 158690058 | - | 3 | intron | LOC141407091,GLRA1 |  |
| 9 | GGGACTCCTCAGCAAGCTGNGG | GGGACTCCcCtGaAAGCTGGTGG | chr6 | 5808510 | + | 3 | intron | ICE1 |  |
| 10 | GGGACTCCTCAGCAAGCTGNGG | tGGACTCCTCAGgAgGCTGTGGG | chr6 | 176596883 | + | 3 | intron | DOCK2 |  |
| 11 | GGGACTCCTCAGCAAGCTGNGG | GGGcCTCCTgAGCtAGCTGAAGG | chr6 | 177189152 | - | 3 | intron | KCNIP1,KCNMB1 |  |
| 12 | CATTAACATCAGAGAGAGAANGG | CATTAAaAagAGAGAGAGAATGG | chr7 | 99299665 | + | 3 | exon | GPR33 |  |
| 13 | CATTAACATCAGAGAGAGAANGG | CAgTcACATtAGAGAGAGAAGGG | chr9 | 69309127 | + | 3 | intron | KCNMA1 |  |
| 14 | GGGACTCCTCAGCAAGCTGNGG | GGGACTCCTCAGttAcCTGCAGG | chr11 | 110351739 | - | 3 | intron | C11H12orf42 |  |
| 15 | GGGACTCCTCAGCAAGCTGNGG | GGGAgTCCTCAGgAAGCaGCAGG | chr12 | 95559833 | + | 3 | intron | TYW5 |  |
| 16 | GGGACTCCTCAGCAAGCTGNGG | tGGACTCCTCAGaAAGaTGGGGG | chr14 | 12502349 | + | 3 | intron | SYT7 |  |
| 17 | GGGACTCCTCAGCAAGCTGNGG | GaGcCTCCTCAGtAAGCTGAGGG | chr14 | 116382823 | - | 3 | intron | DSCAML1 |  |
| 18 | CATTAACATCAGAGAGAGAANGG | CcTgAAaATCAGAGAGAGAAAGG | chr16 | 7243307 | + | 3 | intron | DLG4 |  |
| 19 | CATTAACATCAGAGAGAGAANGG | CtTTtACATCAGtGAGAGAAAGG | chr16 | 69265955 | + | 3 | intron | MARCHF10 |  |

**Table. S4 |List of primers used in off-target sequencing.** The number of primer corresponds to the number in table S3.

| Primer | sequence | PCR product length |
| --- | --- | --- |
| test1-F | TGGAAAAGTTAGAGAACAAGGGT | 619bp |
| test1-R | GCCCAGAAGGAAAGGAAGGG |  |
| test2-F | AGCAACAACAAACCAACCTTCT | 792bp |
| test2-R | AGCCCATTGGCTTTCCTTCT |  |
| test3-F | TGCTCACAGTGCCAGTTTCA | 772bp |
| test3-R | TCATGTGCCTTGCTGGACTT |  |
| test4-F | GTGGTGGAACTGAGGGGATG | 762bp |
| test4-R | CAGAGCAACTCCTGTAGGGC |  |
| test5-F | TCTTGAGAATGTGCTGGGCT | 603bp |
| test5-R | GCACACTGTACCTCCGGAAA |  |
| test6-F | TGACAGCCAGCAACAGTGAT | 722bp |
| test6-R | GCCAAAAACCCATGAGGCAG |  |
| test7-F | CCGAGCCCTTTGTCAGTTCT | 791bp |
| test7-R | CGTGGAGGCTCAATAATGCC |  |
| test8-F | GGGCCTACTGAGTGTTTCCC | 620bp |
| test8-R | AGGTTTGGGGAGGTAGAGTTG |  |
| test9-2F | AGAAAAAGGCAACCTCACAGGTAA | 601bp |
| test9-2R | TCCTGATGGGACCCAAGATT |  |
| test10-F | GGGTGGTCATAGCTGCATGT | 746bp |
| test10-R | GCATGATGAAGGGACAGGCT |  |
| test11-2R | GGTACAGCGGGGACTTGAC | 925bp |
| test11-2F | CTTCTCCCGGATGGGAACTT |  |
| test12-F | CCAGAGCGACACAGACAAGT | 717bp |
| test12-R | TATTTCTTGAGGGGACGGGG |  |
| test13-F | CTGGGGCCTCAGAGACTACT | 642bp |
| test13-R | GGTGAAGGCAGTCACCTTTT |  |
| test14-F | TCTGGCCATGTTCACCCATC | 665bp |
| test14-R | ATCTGCCATGCAACCATCAG |  |
| test15-F | AGCAAGTGAGGAAAACCTGGA | 601bp |
| test15-R | CTGACCGTCACTCCTCAACC |  |
| test16-F | GAACAGCCTCACCACCTCAA | 755bp |
| test16-R | AATCTCGGTGCTGTGTGGAG |  |
| test17-F | AACAAGTTGGCCGCAATGAC | 789bp |
| test17-R | TCCCTTTCTGGAGGAAGAAGC |  |
| test18-F | TGAAGTGACACCCCTTCAGC | 608bp |
| test18-R | TGGGAAAAATCTGAGGCAGC |  |
| test19-F | ACCCCAGAGGGGATCAGTTT | 693bp |
| test19-R | GTAAAGCCCCCTGAAGGTCC |  |

**Table. S5 |Quantification of the self-injurious behavior and stereotyped behaviors in F0 macaques.**

| Self-injurious behavior | WT | MUT |
| --- | --- | --- |
| Mean (min in 12-hr trail) | 0 | 12.58 |
| 95th pencentile | 0 | 46.05 |
| Trail ratio exceeding WT’s 95th pencentile | 0/12 | 9/18 |
| Stereotyped behavior | WT | MUT |
| Mean (min in 12-hr trail) | 6.21 | 43.38 |
| 95th pencentile | 15.96 | 81.28 |
| Trail ratio exceeding WT’s 95th pencentile | 1/12 | 14/18 |

**Table. S6 |Quantification of the self-injurious behavior in F1 macaques.**

| Self-injurious behavior | WT | HET |
| --- | --- | --- |
| Mean (sec in 30-min trail) | 3.74 | 61.20 |
| 95th pencentile | 15.57 | 249.04 |
| Trail ratio exceeding WT’s 95th pencentile | 2/32 | 14/26 |

**Table. S7 |Summary of the behavior abnormities in F0 and F1 DISC1 mutant macaques.**

|  | M1 | M2 | M3 | M4 | HET1 | HET2 |
| --- | --- | --- | --- | --- | --- | --- |
| Stereotype | n |  |  | n | n | n |
| Self-injurious behavior | n | n |  |  |  |  |
| Exploration |  |  | n | n |  |  |
| Relaxed sleep |  |  |  | / |  | n |

Note: upward arrow, increase; downward arrow, decrease; “n”, normal; “/”, not analyzed.
