# Supplementary material for: DISC1 mutant macaques capture behavioral and neural hallmarks of psychiatric disease
Source: Natl Sci Rev. 2026 Mar 28;13(8):nwag200. doi: 10.1093/nsr/nwag200 (PMC13166873; doi:10.1093/nsr/nwag200)
Supplement: nwag200_Supplemental_Files [file nwag200_supplemental_files.zip › Supplementary methods.doc]

Supplementary Methods

**Generation of Crispr-cas9 *Disc1* mutant monkeys**

Selected gRNAs targeting exon 9 of macaque *Disc1* and cas9 mRNA were cloned separately into the pT7 expression plasmid and then in vitro transcribed using MEGAshortscript T7 kit (Thermo Fish Scientific). The products were purified using MEGAclear kit (Ambion). Cas9 mRNA (100 ng) mixed with sgRNA (25 or 50 ng each sgRNA) was injected into one cell-stage macaque embryo with IM-300 microinjecting system (Tritech Research). After injection, the embryos were cultured in Hamster Embryo Culture Medium 9 until 2-4 nuclear stage and were transferred to the oviducts of surrogates.

**Genotyping of mutant monkeys and off-target analysis**

To genotype *DISC1* mutation in newborn macaques, we cut a small epithelial tissue from the top of the ear for genotyping with a pair of primers targeting macaque *DISC1* exon9 (forward 5’-CCAGGTTCTTTCCCCAGAGGACT-3’, reverse 5’- CCGTGACACTCTTCACAGTGACG-3’). Tissues from dead macaques were also collected and incubated in lysis buffer. Genome extraction kits (TIANGEN, lot: A0402B) were used to extract DNA. PCR products were purified and cloned into pMD19-T plasmid, which was later transformed into competent Escherichia coli and plated onto ampicillin selected plates. 20 individual clones one plate were picked up randomly and plasmid DNA was sequenced (Sanger sequencing, GENEWIZ) to examine different types of indels and their percentage. The 5 bp insertion of heterozygotes was also detected by PCR using two pairs of primers with one primer containing the insertion sequence (pair 1: forward 5’-CCAGGTTCTTTCCCCAGAGGACT-3’, reverse 5’-CTCCTCGGTCTTTAAGGTCT-3’; pair 2: forward 5’-AGA CCTTAAAGACCGAGGAG-3’, reverse 5’-CCGTGACACTCTTCACAGTGAC G-3’).

Cas-OFFinder (http://www.rgenome.net/cas-offinder）was used to predict the potential off-target site. 19 predicted sites with fewer than 3 mismatches (type: intron or exon) were further verified with PCR and sequencing using DNA from 17 *DISC1* mutant macaques. The off-target analysis results and related primer information are summarized in Tables S3 and S4.

**Western blot**

46m region of forebrain cortex was cut from frozen brain of WT7, WT10, WT12, M5, HET4 and HET6 and stored at −80◦C until use. Approximately 100 mg tissues were homogenized and lysed in Rippa lysis buffer (Beyotime P0013B) with 1x protease inhibitor cocktail (Bimake B14001). Protein levels were standardized using BCA protein quantification kit (YEASEN 20201ES86) before being boiled in SDS-PAGE loading buffer (Beyotime P0015F). Protein samples (100 μg) were resolved in a 12.5% SDS-polyacrylamide gel then transferred to polyvinylidene difluoride membrane, followed by standard protocols for western blot with primary antibodies: mouse anti-DISC1 (Santa Cruz sc-365591), mouse anti-actin (Merck MAB1501); and secondary antibody: goat anti-mouse IgG HRP conjugate (Beyotime P0612).

**Behavioral assessment**

Each macaque worn a commercial accelerator recorder (QUANLAN) in their collar for one week in the home cage. The data was post-processed using a custom MATLAB R2022a (MathWorks Inc., Natick, MA, USA) program. A camera was set in front of each home cage providing 24-hr recording. The macaques were transferred to a large novel cage (3m x 2m x 2m) in a quiet empty room. For locomotion trials, macaques stayed individually in the cage and recorded with a camera for 5 days (30-min per day with the deletion of the first 5 min and the last 5-min). For social interaction, 2 macaques (2 WT or 1 WT vs. 1 mutant) were put in a novel cage and recorded for 5 days (1 hr per day). For the human intruder test, the macaques were transported individually into the novel cage and were given 12 min to acclimatize. One unfamiliar human intruder came into the room and stood 1.5 meter before the cage without making any eye or body contact for 5 min and left the room. After 5 min, the same human intruder came back to the room and stood at the same position but now making direct eye contact with the monkey for 5 min. Then the intruder left the room for 12 min. The F0 generation took the locomotion and social interaction trial at 30M and 46M. The F1 generation took the locomotion and human intruder trial at 18M.

The home cage videos were manually analyzed by counting the times and duration of stereotyped (repeated behaviors for more than 3 times continuously) and self-injured behaviors. The sleep states were classified by times of body movement in one minute (Awake phases: 0-1 movement, transitional sleep phase: 2-3 movement, relaxed sleep phase: more than 3 movements or eyes open). We used markerless AI-tracking system (DeepLabCut) [56] to trace the routes and calculate the distance and duration of locomotion. For social interaction, we classify the behaviors into affiliative, aggressive and submissive behaviors.

**MRI acquisition**

Brain structures of DISC1 mutant macaques (4Y) and WT macaques at the same age were analyzed using Magnetic Resonance Imaging T1 and T2 Scan using Siemens Tim Trio 3T (TR 2200ms, TE 2.8 ms). The results were analyzed using NMT2.1 as the hierarchical atlas. We used a modified code from MACAQUE_DEMO_2.0 demo [41] including skull stripping, motion correction, animal_warpe and nonlinear transform to the altas.

**ELISA and metabolomics**

The blood (1 ml) and cerebrospinal fluid (200μl) samples were taken from monkeys. The blood samples were centrifuged for 10 min at 3000 rpm and the supernatants were placed on dry ice for targeted metabolomics analysis (APPLIED PROTEIN TECHNOLOGY) or ELISA experiments to detect the concentrations of 5-HIAA (Shanghai Jining Shiye, JN5800971), and DA (Shanghai Jining Shiye, JN844242).

**EEG recording**

In the EEG recording experiment, we used a passive auditory stimulation paradigm by presenting tones with different intensities in a randomized design (80% regular: 60 dB; 20% irregular: 85 dB) to monkeys in a sound-isolated operating room with breath, heart rate and body temperature monitoring. The sound stimulus lasted 100 ms in each trial. Tones were presented using two speakers close to the monkey’s ears. The monkeys were anesthetized using zoletil@50 (0.8 ml/5kg, VIRBAC) and atropine sulfate injection (0.6 ml/5kg, Shanghai Quanyu Biotechnology company). EEG was recorded continuously using 64-channel NeuroScan SynAmps system (Compumedics NeuroScan, Australia) combined with Ag/AgCl disposable surface electrodes using an electroencephalography cap (Boruikang company). The impedance for all electrodes was monitored and settled below 5 kΩ for data collection. EEG data was analyzed using the FieldTrip toolbox using MATLAB. ERPs were calculated for both regular and irregular conditions after referencing, notch filtering, band-pass filtering (0.5 - 30 Hz) and artifact rejection. Then, the MMN was calculated as the difference in ERP between two conditions.

**Fluoxetine treatment**

M3 was treated with fluoxetine hydrochloride (20 mg/tablet, approved for human use; Patheon France, lot: 23340A) at 1.25 mg/kg per day for one week, followed by 2.5 mg/kg per day for eleven weeks. Fluoxetine powder was mixed into apples for oral administration. The animal’s home-cage behaviors were monitored daily via video recording one week after initiating fluoxetine treatment and for one month following cessation of the drug.

**snRNA-seq**

About 1g brain tissue from 46 m (prefrontal cortex) was taken from M2, M6 and two age and gender matched WT controls. Samples were subjected to single nuclei RNA-sequencing using 10x Genomics 3’mRNA single cell v3.1 kit on illumina novaseq platform (Majorbio). The data was analyzed with cellranger and scanpy. Briefly, we firstly used cellranger mkref to creat the reference genome (GCF_037993035.1_T2T-MFA8v1.0), count and process the data. Then, we used scanpy to obtain the umap and cell types, followed by GO and GSEA analysis of differential expressed genes in specific cell types.

**Visual-proprioceptive multisensory integration testing**

Monkeys were seated in a customized monkey chair allowing for right arm extension, and positioned in front of a custom virtual-reality visual display. The right arm was hidden from direct view, however its reflection in a mirror was captured by a CCD camera visualized via a high-resolution projector onto a rear-projection screen. As the monkeys viewed the horizontal mirror suspended between the screen and the table, the virtual arm image appeared to be their real arm on the table. The arm image and visual target were generated and manipulated using the OpenCV graphics libraries in C++ (Visual Studio 2010, Microsoft Co., WA, USA). Hand position was tracked and recorded using a CinePlex Behavioral Research System (Plexon Inc., TX, USA) at a sampling rate of 80 Hz. A color tracking marker was applied to the nail of middle finger and was rendered invisible by adjusting light exposure settings of the camera.

The monkeys were trained to report their proprioceptive arm location by reaching for a target in a visual-proprioceptive integration task. To initiate a trial, the monkeys placed their hand on the starting point (a blue dot, 1.5 cm diameter) and held it stationary for 1000 ms; during this period, they were instructed not to move. Subsequently, the starting point disappeared for 500 ms (the preparation period). Following this period, the reaching target appeared, serving as the ‘go’ signal. The monkeys then had to reach the target within 2500 ms and hold their hand within the target area for 500 ms to receive a juice reward. Any arm movement during this hold period automatically terminated the trial. The intertrial interval (ITI) was 1500 ms, during which the visual scene was blank; after the ITI, the monkey could initiate the subsequent trial. The starting point was fixed 15 cm from the monkeys’ shoulder. The reaching target was selected randomly on each trial from one of five possible positions located along a 40-cm radius arc centered at the shoulder: specifically at 0° (straight ahead), ±10° or ±20° relative to the shoulder midline. To prevent memorization, a ±4° jitter was applied to the nominal target position on each trial. The monkeys performed a visual-proprioceptive (VP) congruent block and a proprioception-only (P) block per day, with six days in total. In the VP task the visual arm remained congruent with the proprioceptive arm throughout the entire trial. Conversely, in the P task visual arm information was removed after the preparation period. To ensure the monkeys correctly performed the task, the target area was set from −5° to +5° around the target. Trials with a response outside the target zone were removed from the offline analysis.

**Statistical analysis**

We used the Student's t-test and Tukey’s multiple comparisons test for behavior, brain volume comparation and one-way ANOVA for multisensory perception abnormality. Statistical tests were conducted using Prism (GraphPad)

**R****EFERENCES**

56.	Mathis, A., et al., *DeepLabCut: markerless pose estimation of user-defined body parts with deep learning.* Nat Neurosci, 2018. **21**(9): p. 1281-1289.
